# Distribution of 5-Hydroxymethylcytosine in Different Human Tissues

**DOI:** 10.4061/2011/870726

**Published:** 2011-06-09

**Authors:** Weiwei Li, Min Liu

**Affiliations:** R&D Division, Epigentek Group Inc., 110 Bi County Boulevard, Suite 122, Farmingdale, NY 11735, USA

## Abstract

5-hydroxymethylcytosine (5-hmC) is a modified form of cytosine recently found in mammalians and is believed, like 5-methylcytosine, to also play an important role in switching genes on and off. By utilizing a newly developed 5-hmC immunoassay, we determined the abundance of 5-hmC in human tissues and compared 5-hmC states in normal colorectal tissue and cancerous colorectal tissue. Significant differences of 5-hmC content in different tissues were observed. The percentage of 5-hmC measured is high in brain, liver, kidney and colorectal tissues (0.40–0.65%), while it is relatively low in lung (0.18%) and very low in heart, breast, and placenta (0.05-0.06%). Abundance of 5-hmC in the cancerous colorectal tissues was significantly reduced (0.02–0.06%) compared to that in normal colorectal tissues (0.46–0.57%). Our results showed for the first time that 5-hmC distribution is tissue dependent in human tissues and its abundance could be changed in the diseased states such as colorectal cancer.

## 1. Introduction

DNA methylation is an epigenetic modification which is catalyzed by DNA cytosine-5-methyltransferases (DNMTs) and occurs at the 5-position (C5) of the cytosine ring, within CpG dinucleotides. DNA methylation is essential in regulating gene expression in nearly all biological processes including development, growth, and differentiation [1–3]. Alterations in DNA methylation have been demonstrated to cause the changes in gene expression. For example, hypermethylation leads to gene silencing or decreased gene expression while hypomethylation activates the genes or increases gene expression. Region-specific DNA methylation is mainly found in 5′-CpG-3′dinucleotides within the promoters or in the first exon of genes, which is an important pathway for the repression of gene transcription in diseased cells. 

Very recently, a modified nucleotide, 5-hydroxymethylcytosine (5-hmC) was detected to be abundant in mouse brain and embryonic stem cells [4–6]. 5-hmC is a hydroxylated and methylated form of cytosine and was first seen in bacteriophages in 1952 [7]. In mammals, it can be generated by a TET protein-mediated reaction [5]. The exact function of 5-hmC in epigenetics is still a mystery today. However, a line of evidence showed that conversion of 5-mC to 5-hmC greatly reduced affinity of MBD proteins to methylated DNA [8], and 5-hmC accounts for roughly 40 percent of the methylated cytosine in Purkinje cells and is also specifically localized in CpG regions [4]. Thus, 5-hmC might also play an important and different role in regulation of DNA methylation, chromatin remodeling, and gene expression in a tissue-, cell-, or organ-specific manner.

Because of the presence of 5-hmC in DNA with unclear functions in gene regulation and the discovery of the TET enzymes that produce 5-hmC, it is considered necessary to determine the distribution of this modified DNA base in different human tissues. It would be particularly important to identify the changes of 5-hmC abundance in different disease states in human tissues, which is necessary not only for reevaluating the existing methylation datasets, but also for gaining correct information on epigenetic regulation of physiological and pathological process. Tissue distribution of 5-hmC in mouse was described [9]. However, to our knowledge, there is currently no information available about the status of 5-hmC or hydroxymethylated DNA in human tissues, specifically the distribution of 5-hmC in different tissues and its alterations in diseased states. By utilizing a 5-hmC immunoassay recently developed by us, we quantified the content of 5-hmC in human tissues and compared 5-hmC states in normal colon tissue and cancerous colon tissue. We found that the content of 5-hmC varies in different human tissues and is significantly decreased in cancer compared to normal tissues.

## 2. Materials and Methods

### 2.1. DNA Isolation from Tissue Samples and Cell Lines

The following DNA samples were obtained from BioChain (Hayward, Calif, USA): (1) frozen tissues of human brain, lung, heart, liver, kidney, colorectum, and colorectal cancer; (2) frozen tissues of mouse brain. The human placenta DNA was obtained from Bioline (Taunton, Mass, USA). The following DNA samples were prepared using FitAmp Blood and Cultured Cell DNA Extraction Kit (Epigentek, NY, USA) from Hela cervical cancer cell line, HCT116 colon cancer cell line, SW620 colon cancer cell line, and AN3CA endometrial cancer cell line. The concentrations of all DNA samples are measured with spectrophotometer and confirmed with a PicoGreen dsDNA quantitation kit (Invitrogen, Calif, USA). A 260/280 ratio of all DNA samples is greater than 1.8.

### 2.2. Generation of Reference DNA Fragments Containing Cytosine, 5-mC, and 5-hmC

DNA fragments containing cytosine, 5-mC, or 5-hmC were amplified by PCR using a region of hMLH1 containing promoter and exon1. A starting amount of 1 ng of human placenta DNA was used to generate 693 bp DNA amplicons by PCR reactions with a reaction buffer containing 0.2 mM of each dNTP (or 5mdCTP or 5hmdCTP in place of dCTP) and Phire hot start polymerase (Finnzymes, Mass, USA). PCR reactions in 20 *μ*L reaction volumes were carried out according to the manufacturer's instructions using the forward primer 5′-GTCCAAGGCAAGAGAATAGG-3′ and the reverse primer 5′-AGCCAATAGGAGCAGAGATG-3′. To effectively remove unmodified DNA templates from the final products, subsequent PCR amplifications were performed using 1 *μ*L of first round PCR products in 20 *μ*L of reaction volume under the same reaction conditions with different primers to generate 357 bp DNA products. These DNA products contain about 25% cytosine (umDNA), 25% 5-mC (mDNA), and 25% 5-hmC (hmDNA), respectively. PCR products were then run on a 1.5% agarose gel to confirm the correct length and purified by a sodium acetate/ethanol precipitation method.

### 2.3. 5-hmC Immunoassay

200 ng of DNA was mixed with 100 *μ*L of DNA binding solution and then added into the assay wells of microplates. The solution was incubated at 37°C for 90 min to allow DNA binding onto the assay wells tightly. The wells were washed 3 times with PBS-T, and anti-5-hydroxymethylcytosine polyclonal antibody (Epigentek, NY, USA) was added into the wells at 1 *μ*g/mL and incubated at room temperature for 60 min. After washing 3 times, biotin-conjugated antirabbit antibody (Pierce) at 0.2 *μ*g/mL was added and incubated at room temperature for 30 min. After washing with PBS-T 4 times, the signal enhancing solution containing avidin-peroxidase complex was added and incubated for 30 min. After washing with PBS-T 4 times, TMB (100 *μ*L per well) was added and incubated at room temperature for 10 min, and then 50 *μ*L of 1 M HCl per well was added to stop the enzymatic reaction. The reference DNA fragments containing 5-hmC and 5-mC were used as the positive standard and negative control, respectively. The absorbance end point (optical density, OD) was read on a Max kinetic microplate reader (Molecular Devices, Calif, USA). The amount of 5-hydroxymethylcytosine is proportional to the OD intensity measured. After subtracting negative control readings from the readings for the sample and the standard, the value of 5-hydroxymethylcytosine for each sample was calculated as a ratio of sample OD relative to the standard OD.

### 2.4. 5-mC Quantification

The content of 5-mC or methylated DNA was quantified using a MethylFlash Methylated DNA Quantification Kit (Epigentek, NY, USA) according to the manufacturer's instruction.

### 2.5. Data Analysis

Data are reported below as mean ± SD. Slope of the standard curve is determined using linear regression, and the percentage of 5-hmC in the total DNA is calculated using the following formula:

(1) $$5\text{-hmC\%}=\frac{\text{Sample}\;\text{OD}-\text{Negative}\;\text{Control}\;\text{OD}}{\text{Slope}\times \text{Input}\;\text{DNA}\;\text{Amount}}\times 100\text{\%}.$$

## 3. Results

### 3.1. Sensitivity and Specificity of 5-hmC Immunoassay

**Figure 1:**
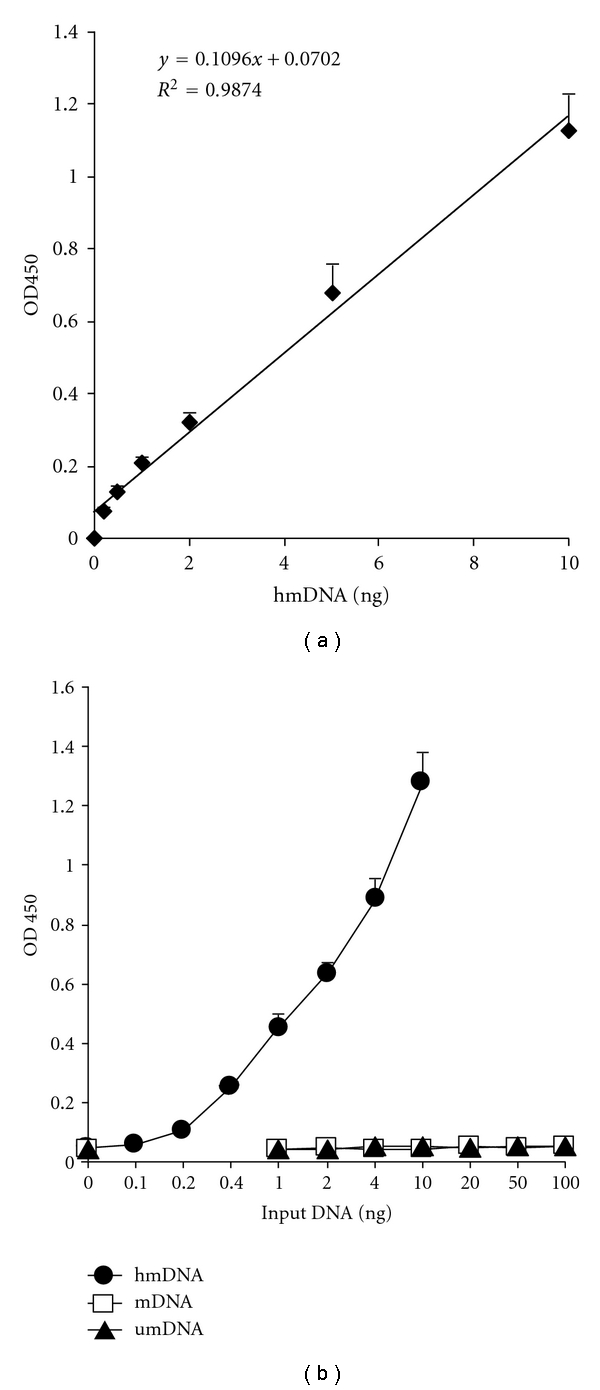
Sensitivity and specificity determination of 5-hmC by the immunoassay. (a) Linear relationship between the absorbance and amount of hmC-containing hmDNA reference fragment (b).

To determine if signal intensity is proportional to the amount of 5-hmC in this immunoassay, the reference hmDNA fragments were added in the assay wells at different concentrations. As shown in Figure 1(a), the OD values were increased with the amount of hmDNA. Dose-response curve is linear in the concentration range of 0.2 ng to 10 ng. To test the sensitivity and specificity of the assay, different amounts of umDNA, mDNA, and hmDNA were used in the assay. The results (Figure 1(b)) showed that the absorbance signal was linearly increased with the input amount of hmDNA, and OD values were detected from the concentration point as low as 0.1 ng of hmDNA, while absorbance intensity generated from umDNA and mDNA was not increased with increased amount. Importantly, even if the amount of mDNA was increased to 100 ng, 10-fold higher than the maximal amount of hmDNA, the absorbance intensity of mDNA is still at the background level (blank control with no DNA). This demonstrates that this assay allows discrimination between 5-hmC and 5-mC to be greater than 1 : 1000.

### 3.2. Distribution of 5-hmC in Different Human Tissues and Cell Lines

**Figure 2:**
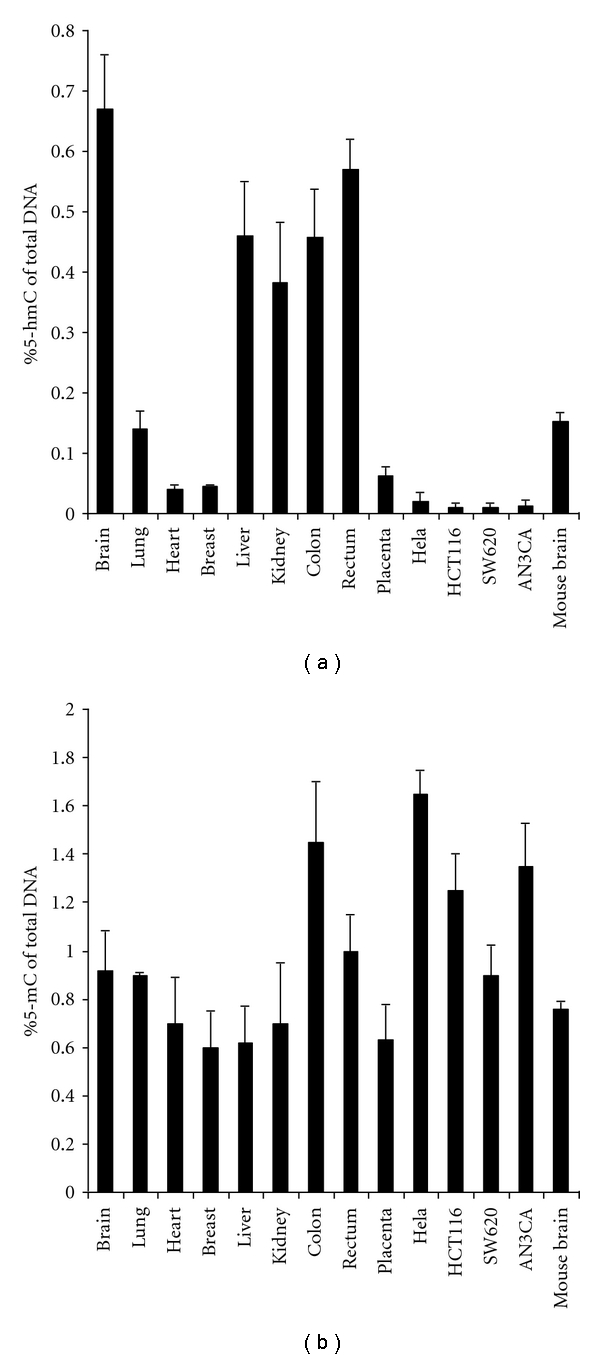
Quantification of 5-hmC and 5-mC in genomic DNA isolated from human tissues. (a) 5-hmC contents. The data are average values ± standard deviation from 3 different assays. (b) 5-mC contents. The data are average values ± standard deviation from 3 different assays.

Utilizing this assay we determined the content of 5-hmC in different human tissues and cell lines. DNA was isolated from 9 normal human tissues of individuals aged from 24 to 78 years old. These tissues include brain, lung, breast, heart, kidney, placenta, breast, colon and rectum. DNA was also isolated from 4 cell lines including Hela cervical cancer, ANCA3 endometrial cancer, HCT116 colon cancer, and SW620 colon, cancer cells. As shown in Figure 2(a), significant differences of 5-hmC content in different tissues were observed. For example, the percentage of 5-hmC measured in brain is 0.67%, which is about 13-fold higher than that in the heart (0.05%). It was also observed that 5-hmC was abundant in kidney (0.38 %), colon (0.45%), rectum (0.57%), and liver (0.46%) tissues. In contrast, 5-hmC content was relatively low in lung (0.14%), and very low in breast (0.05%) and placenta (0.06%). Interestingly, little 5-hmC content was detected in all 4 cell lines (<0.02%). We also measured the 5-hmC amount in mouse brain tissues. The known 5-hmC content in mouse brains was reported early [4, 6, 9, 10]. The amount of 5-hmC measured by our assay in mouse brain was 0.15% of total DNA, which is similar to that analyzed by LC-MS (0.13–0.15% of total DNA) [6, 9] or by radioactive hmC glucosylation assay (0.24% of total DNA) [10]. Thus, this result would provide supporting evidence to indicate that the 5-hmC contents measured in human tissues are accurate. We further measured the 5-mC contents in these tissues in order to compare the 5-mC and 5-hmC distribution patterns in the same tissues. As shown in Figure 2(b), the 5-mC content of all tissues ranged from 0.6% to 1.5%, which makes the differences of the 5-mC amount in these tissues be only 1- to 2.5-fold. Similarly, the differences of 5-mC amount in 4 the cell lines are also not significant (0.8%–1.6%).

### 3.3. 5-hmC Is Decreased in Cancerous Tissues

**Figure 3:**
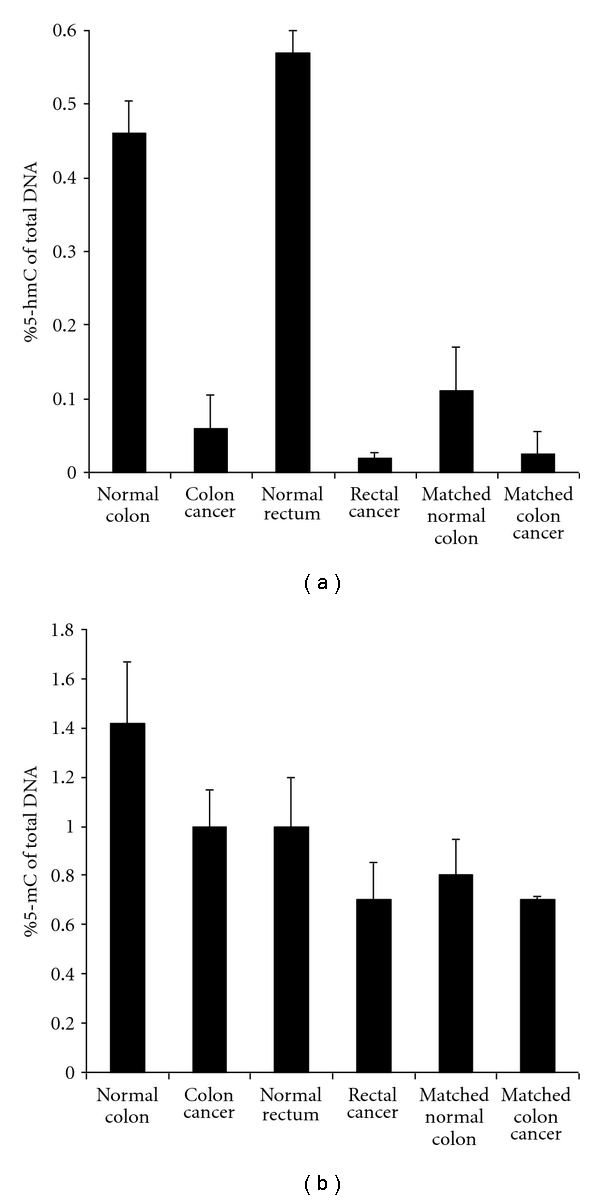
Contents of 5-hmC and 5-mC measured from normal colon and cancerous colon tissues. (a) 5-hmC contents. The data are average values ± standard deviation from 3 different assays. (b) 5-mC contents. The data are average values ± standard deviation from 3 different assays.

Because tissues-specific distribution of 5-hmC was observed, it is interesting to determine if 5-hmC content will be changed when tissue/cells are in diseased states such as cancer.  We thus quantified the 5-hmC content in cancerous colorectal tissues from two individuals with moderately differentiated colon or rectal carcinoma. Compared to 5-hmC contents of normal colon and rectal tissues (0.46% and 0.57%, resp.), the percentage of 5-hmC in colon cancer and rectal cancer measured was 7.7-fold (0.06%) and 28-fold (0.02%) less, respectively. To further confirm if 5-hmC is really decreased in cancerous colorectal tissues, we examined the 5-hmC contents in another colon cancer (moderately differentiated) and the matched adjacent normal colon tissues from a 56-year-old individual. The results showed that the percentage of 5-hmC in cancerous tissue was only 0.025%. In contrast, 5-hmC content was 4.4 times more (0.11%) in adjacent normal colon tissue, though it was still much less than that obtained from normal colorectal tissues from healthy individuals (Figure 3).

## 4. Discussion

In this study, we described the distribution of 5-hmC in various human tissues. Unlike the content of 5-mC, which is only 1–2.5-fold different in tested human tissues, 5-hmC content showed a 13-fold greater difference in different tissues. Brain tissue contains the highest level of 5-hmC, and liver, colorectum and kidney present relatively high abundance of 5-hmC. Generation of 5-hmC in mouse cells was believed to be mainly due to an enzymatic conversion of 5-mC to 5-hmC by TET1 protein or TET protein family [5, 11]. 5-hmC contents in mouse ES cells seem also corrected with expression of TET protein family (TET1-3) [10]. However, tissue-specific distribution of 5-hmC in human tissues that we observed may not be explained only by this mechanism as TET proteins were strongly expressed in heart and placenta tissues that contain extremely low 5-hmC amount; and there was no correlation between high 5-hmC content and low 5-mC level in each type of tissue. The mechanisms of tissue-specific distributions of 5-hmC in human tissues still need further exploration. 

It is particularly interesting to find that 5-hmC is significantly reduced in cancerous colorectal tissues and even decreased to an undetectable level in colon cancer cell lines. The detection with increased samples (38 colon cancer tissues and 8 normal colon tissues) further confirmed 5-hmC content in colon cancer is 4-fold lower than that in normal colon tissues (data not shown). These results suggested that 5-hmC may negatively regulate cancer formation and development at least in colorectal tissues. Numerous evidences showed that methylation-mediated silencing of tumor suppression and apoptosis genes is involved in cancer formation and progression. 5-hmC has been shown to prevent DNA methylation by blocking the maintenance DNA methyltransferase (DNMT1) from methylating DNA containing 5-hmC [12] and involves the maintenance of gene expression by turnover of methylation [11]. Thus, it is possible that the 5-hmC reduction in cancerous colorectal tissues damages the reactivation of these genes through 5-hmC-mediated methylation turnover, which would help the cancer cells to escape from tumor suppression and apoptosis caused by products of these genes. How 5-hmC is decreased in cancerous colorectal tissues is still unclear. It is worth exploring whether it could be due to decreased 5-hmC production resulting from a decrease in TET proteins or mutation of these proteins [13, 14] and whether it could be more likely due to an increase in enzymatic removal or conversion of 5-hmC in cancer tissues. In addition, 5-hydroxymethylcytosine DNA-glycosylase that removes 5-hmC which was found in mammalian tissue [15] may also abundantly exist in cancerous colorectal tissues and contribute to a decrease in 5-hmC.

## 5. Conclusion

To the best of our knowledge, this is the first paper of 5-hmC distribution in different human tissues and 5-hmC status in solid tumor. It warrants on further investigation of 5-hmC states in colorectal cancer by using a large quantity of samples. It would also be necessary to determine 5-hmC states in other cancer types derived from 5-hmC abundant tissues such as brain and liver and more widely in other epigenetic-associated diseases such as neurodegenerative disorders. 5-hmC determination would help to further understand methylation/demethylation regulation in the formation and development of epigenetic-associated diseases, thereby benefiting diagnostics and therapeutics at early stages of these diseases.
